# Ambulatory searching task reveals importance of somatosensation for lower-limb amputees

**DOI:** 10.1038/s41598-020-67032-3

**Published:** 2020-06-23

**Authors:** Breanne P. Christie, Hamid Charkhkar, Courtney E. Shell, Christopher J. Burant, Dustin J. Tyler, Ronald J. Triolo

**Affiliations:** 1 Department of Biomedical Engineering, Case Western Reserve University, Cleveland, OH USA; 2 Louis Stokes Cleveland Deptartment of Veterans Affairs Medical Center, Cleveland, OH USA; 3 Department of Biomedical Engineering, Lerner Research Institute, Cleveland Clinic, Cleveland, OH USA; 4 School of Nursing, Case Western Reserve University, Cleveland, OH USA

**Keywords:** Somatic system, Sensory processing, Biomedical engineering

## Abstract

The contribution of somatosensation to locomotor deficits in below-knee amputees (BKAs) has not been fully explored. Unilateral disruption of plantar sensation causes able-bodied individuals to adopt locomotor characteristics that resemble those of unilateral BKAs, suggesting that restoring somatosensation may improve locomotion for amputees. In prior studies, we demonstrated that electrically stimulating the residual nerves of amputees elicited somatosensory percepts that were felt as occurring in the missing foot. Subsequently, we developed a sensory neuroprosthesis that modulated stimulation-evoked sensation in response to interactions between the prosthesis and the environment. To characterize the impact of the sensory neuroprosthesis on locomotion, we created a novel ambulatory searching task. The task involved walking on a horizontal ladder while blindfolded, which engaged plantar sensation while minimizing visual compensation. We first compared the performance of six BKAs to 14 able-bodied controls. Able-bodied individuals demonstrated higher foot placement accuracy than BKAs, indicating that the ladder test was sensitive enough to detect locomotor deficits. When three of the original six BKAs used the sensory neuroprosthesis, the tradeoff between speed and accuracy significantly improved for two of them. This study advanced our understanding of how cutaneous plantar sensation can be used to acquire action-related information during challenging locomotor tasks.

## Introduction

Limb loss results in an interruption of sensorimotor coordination. Despite decades of technological advancements, unilateral lower-limb amputees continue to face challenges during ambulation. They typically have decreased balance confidence^1^, increased fall risk^2^, and increased cognitive burden compared to able-bodied individuals^3^. They also adopt a slower, more asymmetrical gait^4–13^ that leads to chronic lower back, hip, and knee pain^14^. These challenges can be attributed to the mechanical properties of prosthetic feet and limited sensory feedback; locomotor navigation requires adjustments that are informed by sensory information. Commercially available prostheses do not have volitional ankle control, and inversion/eversion is limited, if it is available at all. Somatosensory feedback is limited to interactions between the socket and residual limb. Though unilateral lower-limb amputees may benefit from restoring cutaneous plantar sensation, its impact on their locomotion is not well defined.

Cutaneous plantar sensation is an essential component of able-bodied locomotion because it provides information regarding interactions of the foot with the environment. Mechanoreceptors in the skin of the foot sole transduce spatial and temporal information about contact pressures, and indirectly, the moment applied at the ankle joint^15,16^. Balance is impaired when plantar sensation is temporarily eliminated in both feet of able-bodied people, as indicated by increased postural sway^17,18^, a more crouched posture during gait^19^, and more reactive steps needed to restore equilibrium following perturbations^20^. Able-bodied individuals also adopt a more cautious walking pattern when cutaneous plantar sensation is eliminated in just one foot: dorsiflexion is reduced at the beginning of the stance phase of gait, and plantarflexion is decreased during push-off^21^. These locomotor adaptations during unilateral sensory disruption resemble some of the strategies adopted by unilateral lower-limb amputees^11^. When combined with prosthesis designs that more closely resemble the mechanical properties of the intact foot, the restoration of somatosensory information has the potential to decrease locomotor deficits.

Despite this potential, no commercially available prostheses provide natural somatosensory feedback. To our knowledge, just two prior studies have evaluated the functional efficacy of below-knee prostheses equipped with plantar pressure feedback^22,23^. In those studies, an electrical or vibratory stimulus was applied to the skin of the residual limb. Neither study revealed a significant improvement in balance or gait. This was likely influenced by two factors: the mechanism of sensory feedback and the method of functional testing. Both prior studies delivered sensory feedback to the skin of the residual leg, which did not selectively activate the afferent sensory fibers formerly innervating the foot. This imposed a learning curve for associating a substitutive sensory stimulus with pressure on the prosthetic foot, rather than perceiving somatosensory feedback as originating in the missing foot. In a prior study, we demonstrated that electrically stimulating the nerves in the residual limbs of amputees elicited somatosensory percepts that were reported as occurring in the missing foot^24^. In the present study, we employed a sensory neuroprosthesis that modulates stimulation-evoked somatosensation in response to interactions between the prosthesis and the environment. We aimed to evaluate the functional benefits of this sensory neuroprosthesis in a novel ambulatory task that specifically isolated unilateral plantar sensation.

It is difficult to isolate and assess the role of unilateral plantar sensation in locomotion because humans integrate multiple streams of sensory information, such as vision, vestibular inputs, cognitive attention, somatosensory inputs, and proprioception to interpret and respond to the surrounding environment^25,26^. Unilateral lower-limb amputees are missing afferent information from sensory receptors in the lost muscles, tendons, and skin of one leg, but can compensate for minor threats to stability by relying on their remaining resources^27^. In particular, they tend to prioritize information from their intact limbs and visual inputs during locomotor tasks^28,29^. To our knowledge, no existing tests evaluate the role of plantar sensation in one foot while minimizing compensation by vision and/or sensory information from the other foot.

The horizontal ladder walking test is an ambulatory searching task that has been routinely applied in sensorimotor research in rodents^30^, felines^31^, and non-human primates^32^. The test involves walking over a horizontal ladder that has variable inter-rung spacing, which changes after each trial to minimize learning. The feline model, in particular, has been utilized to study the role of somatosensation during locomotion. After the cutaneous sensory nerves below the ankle were severed, felines placed their paws on the ladder rungs less precisely than before denervation^31^. A similar experimental construct had not yet been adapted for bipedal locomotion, which led us to rescale and customize the horizontal ladder test for human participants. We also blindfolded individuals to minimize visual compensation. We conducted this test with volunteers with and without limb loss to characterize the sensitivity of the horizontal ladder test to locomotor deficits.

In this study, our first goal was to quantify how able-bodied individuals and below-knee amputees (BKAs) performed on the horizontal ladder test, since the evaluation had yet to be applied to either population. We hypothesized that able-bodied controls would perform better on the task, demonstrated by faster completion times and fewer foot placement errors. Our second goal was to evaluate a subset of the lower-limb amputees’ performance on the test with a standard prosthesis versus a sensory neuroprosthesis. We hypothesized that receiving somatosensory feedback would improve lower-limb amputees’ task performance to more closely resemble that exhibited by able-bodied individuals.

## Materials and methods

### Research participants

Fourteen able-bodied volunteers (AB01-AB14), eight women and six men, served as a control group. On average, participants were 34 ± 16 years old (mean ± standard deviation) and 1.7 ± 0.08 m tall (see Supplemental Table S1 for more detailed demographics). Six volunteers with unilateral below-knee (trans-tibial) amputations were also enrolled in this study (BKA01-06), and three of them also performed the test using the sensory neuroprosthesis (BKA01-03). All BKAs were male with a mean age of 57 ± 10 years and height of 1.75 ± 0.08 meters (Supplemental Table S2). Four below-knee amputations were caused by trauma, one was caused by vascular disease, and one was caused by skin cancer. None of the BKAs had significant medical history of neuropathy in the intact leg. All amputees were regular prosthesis users and wore their clinically-prescribed personal prostheses for all experiments. Five BKAs wore energy-storage-and-return prostheses and one wore an active prosthesis with the powered ankle turned off (i.e., locked at a neutral angle) during experiments. The Louis Stokes Cleveland Veterans Affairs Medical Center Institutional Review Board and Department of the Navy Human Research Protection Program approved all experimental procedures. Portions of this study involving the sensory neuroprosthesis were conducted under an Investigational Device Exemption obtained from the United States Food and Drug Administration. All participants gave their written informed consent prior to participation in research-related activities, which were designed in accordance with the relevant guidelines and regulations.

### Experimental design

**Figure 1:**
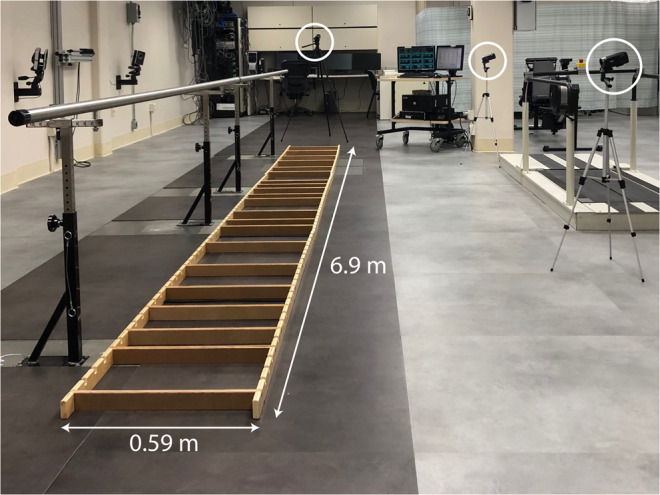
Experimental setup. Able-bodied volunteers and below-knee amputees performed a horizontal ladder rung walking test while blindfolded. Ladder rungs were randomly spaced 19, 28.5, 38, or 47.5 cm apart and the arrangement changed after every trial. Participants used a single handrail that ran alongside the ladder for support. Videos were recorded with three cameras, two alongside the ladder and one at the end.

The testing apparatus was constructed out of wood and measured 6.9 m long by 0.59 m wide, as depicted in Fig. 1. There were 22 ladder rungs in total, each of which was flat on top and 1.9 cm wide. Rungs were randomly spaced 19, 28.5, 38, or 47.5 cm apart. Six different ladder arrangements were used to minimize learning, and participants were blinded to the rung spacing. Each trial consisted of crossing the ladder in one direction, after which participants could take an optional break. The ladder arrangement changed after a participant had crossed the ladder once in each direction. A single handrail that ran along one side of the ladder was available to participants for safety and to assist with maintaining balance and heading. Two video cameras were set up perpendicular to the ladder along its length for sagittal plane views of the participants, and a third video camera was placed at the end of the ladder for an orthogonal coronal plane view. Weights were placed around the ladder to ensure that it did not move during an experiment (not pictured in Fig. 1).

All participants were blindfolded and instructed to: a) walk foot-over-foot, b) step on one rung at a time, and c) not step on the floor or ladder side rails. They were not given any instructions about speed nor told that they would be evaluated based on their pace. Participants wore their own closed-toe shoes. Force-sensing insoles (IEE S.A.; Bissen, Luxembourg) consisting of eight force-sensitive resistor (FSR) cells were placed inside each shoe. Pressure readings from each cell were collected at a sampling rate of 1000 Hz using a tethered system with a data acquisition board. The readings were recorded via Vicon Nexus software (version 2.8.2; Oxford, UK) or MATLAB (MathWorks, Inc.; Natick, MA, USA). MATLAB was used only for participants BKA01-03 because it simultaneously collected pressure data and generated neural stimulation according to the paradigms described later.

Participants performed two practice trials and 16 data collection trials. To evaluate the functional influences of the sensory neuroprosthesis, three of the volunteers with below-knee amputations (BKA01-03) performed additional trials. BKA01-03 performed the task an additional 12 times without stimulation (28 total trials) and 28 times with stimulation-evoked somatosensory feedback. Trials with stimulation-evoked somatosensation were impossible to blind to the participants, but they were randomly interleaved throughout the 28 trials without somatosensory feedback.

### Outcome measures

Primary outcome measures included foot placement accuracy and trial completion time, which were extracted by reviewing video recordings. Accuracy was defined by the number of errors per trial. Errors included: a) missing a rung, b) slipping off a rung, c) simultaneously stepping on a rung and the floor, d) placing the foot on two rungs at once, and e) stepping on a side rail. Participants could perform “searching taps” on the side rail or the floor prior to stepping on a ladder rung without being penalized.

**Figure 2:**
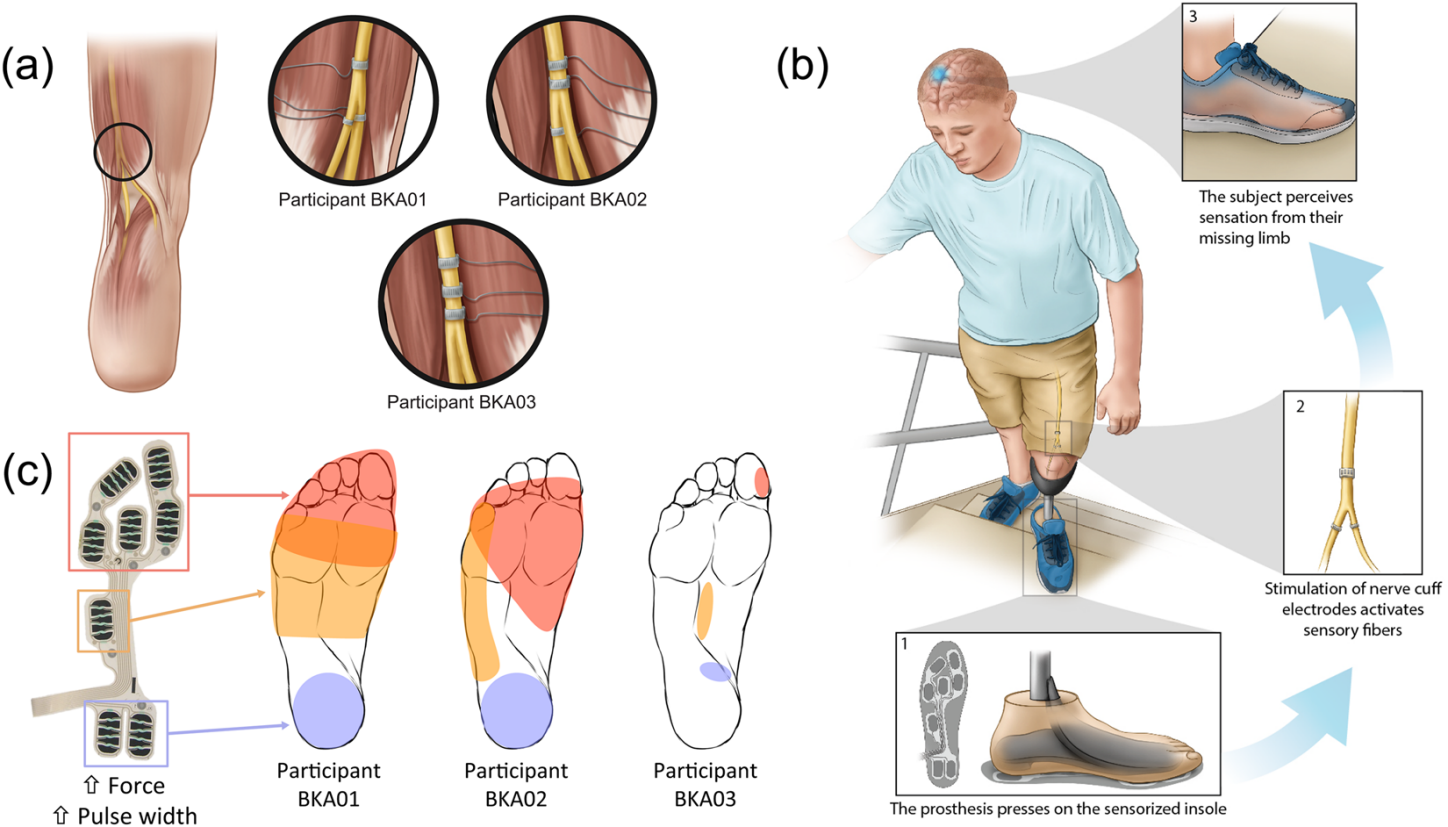
Closed-loop somatosensory neuroprosthesis. (**a**) Location of nerve cuff electrodes for three participants with below-knee amputations. Three 16-contact C-FINEs were implanted around the sciatic, tibial, and common peroneal nerves of Participant BKA01. For participant BKA02, two C-FINEs were implanted around the sciatic nerve and one around the tibial nerve. Three C-FINEs were implanted around the sciatic nerve of participant BKA03. **(b)** Depiction of the closed-loop system. An insole containing eight FSRs is located in the shoe underneath the prosthesis. Pressure on the FSRs proportionally modulates the pulse width of electrical stimulation through corresponding C-FINE contacts to activate sensory fibers associated with the region of applied pressure. Activation of these sensory fibers causes the amputee to perceive sensations as if they originated in the missing limb. **(c)** Perceived locations of percepts felt with the closed-loop sensory neuroprosthesis. When pressure was exerted on the forefoot of the prosthesis, the participants reported that percepts occurred in the shaded red area. Percepts reported by pressing on the midfoot and rearfoot are shown in orange and purple, respectively. Illustrations 2a and 2b are courtesy of the APT Center at the Louis Stokes Cleveland VA Medical Center.

To further explore which factors may contribute to any differences in accuracy and completion time between able-bodied individuals and amputees, we also analyzed foot placement strategy, which was defined as the region of the foot placed on each ladder rung. In this context, foot placement strategy is affected by biomechanics and the presence or lack of volitional ankle control, but potentially also cutaneous sensation. We identified which region of the foot was placed on each rung by simultaneously processing the video recordings and pressure readings. After using video recordings to verify that a step was definitive and on a ladder rung (i.e., not a searching/tapping maneuver or an accidental step on the ground), pressure readings were examined to confirm which region of the foot was placed on the rung. The mean pressure in each FSR cell during a step was calculated over a 200 ms window consisting of 100 data samples before and after the selected time point (+100 ms). For simplicity, we categorized the FSR cells into three groups corresponding to three regions of the foot: forefoot, midfoot, and rearfoot (Fig. 2c). The region with the highest average pressure per FSR cell defined the region of the foot used to step on the ladder rung, thus accounting for the variation in number of FSR cells in each region.

### Closed-loop sensory neuroprosthesis

To deliver neural stimulation, 16-contact Composite Flat Interface Nerve Electrodes (C-FINEs)^33^ were chronically implanted around the residual sciatic, tibial, and/or common peroneal nerves in participants BKA01-03 (Fig. 2a). The internal C-FINEs were connected to an external stimulator^34,35^ by percutaneous leads that exited the skin on the upper anterior thigh. The details of the implant procedure, post-operative care, and percutaneous access to the contacts within each C-FINE are described in our prior work^24^. The sensory neuroprosthesis recipients received an amputation 47 years (BKA01), nine years (BKA02), and eight years (BKA03) prior to implant surgery. The experiments in this study were performed at least five months post-implantation, during which time participants received neural stimulation every 1-4 weeks to characterize the psychometric properties of elicited sensations^24,36,37^.

Stimulation-evoked somatosensation was modulated by pressure applied to the FSRs underneath the forefoot, midfoot, and rearfoot regions of the prosthetic foot (Fig. 2b). For each participant, a subgroup of C-FINE contacts was selected that consistently elicited sensations in the forefoot, midfoot, and/or rearfoot of the missing limb with electrical stimulation (Fig. 2c). The participants reported these regions by repeatedly drawing the locations of the percepts on a diagram of the foot. At the beginning of each experimental session, participants were asked to stand upright without moving as the pressure distribution on the FSRs was captured. They were then asked to apply pressure to different isolated areas of the foot, such as the forefoot or rearfoot, so that the maximum pressure in each region could be determined. In each position, a range of stimulation parameters was mapped to these pressure ranges and used throughout the duration of the session. The perceived magnitude of evoked sensations was scaled proportionally to the amount of pressure applied to the FSRs. This modulation was done by increasing or decreasing stimulation pulse width in real-time while holding pulse amplitude and frequency constant^38^. Pulse width varied between 120-250 μs. This process was repeated at the beginning of each testing session to minimize the influence of confounding factors, such as insole placement within the shoe. More in-depth details about stimulation parameter tuning can be found in Charkhkar *et al*.^39^.

Stimulation waveforms were monopolar, asymmetric biphasic, charge-balanced, cathodic-first pulses with return to a common anode placed on the skin above the ipsilateral iliac crest. Pulse frequency and amplitude were set for each C-FINE contact and held constant throughout each session. Pulse frequency ranged from 20 to 100 Hz between contacts and pulse amplitude ranged from 0.7 to 1.2 mA. Stimulation parameters were set in MATLAB and then sent to a single board computer running xPC real-time kernel (MathWorks, Inc.; Natick, MA, USA), which controlled the external stimulator in real time. An isolator between the xPC target computer and the stimulator ensured optical isolation between the participant and line-powered instruments. Stimulation was limited to a charge density of 0.5 μC/mm^2^ in order to minimize the risk of tissue and/or electrode damage^40^.

### Statistical analysis

To compare the performance of able-bodied individuals versus BKAs, we performed a 16 × 2 repeated measures linear mixed model. This approach increased statistical power because the effective sample became 320 data points (20 participants*16 repetitions) as compared to 20 data points based on the average performance by each individual participant. Repeated measurements on each participant were accounted for by a compound symmetry error covariance structure. With this model, comparisons of the primary outcome measures were made between participant groups.

To determine if there was an effect of gender on the primary outcome measures for able-bodied participants, we performed a two-tailed, two-sample t-test. We also completed linear regressions to determine if there was an effect of age or height. We performed additional linear regressions to evaluate the relationship between completion time and the number of errors for each group of participants. To compare performance of the BKAs with and without stimulation-evoked feedback, each participant served as his own control. One-tailed, paired t-tests compared completion time and the number of errors between sensory conditions, with the hypothesis that each outcome measure would decrease during trials with stimulation-evoked somatosensory feedback. Significance levels of *α* = 0.05 deemed a statistically significant result.

## Results

### Comparison of able-bodied individuals and below-knee amputees

**Figure 3:**
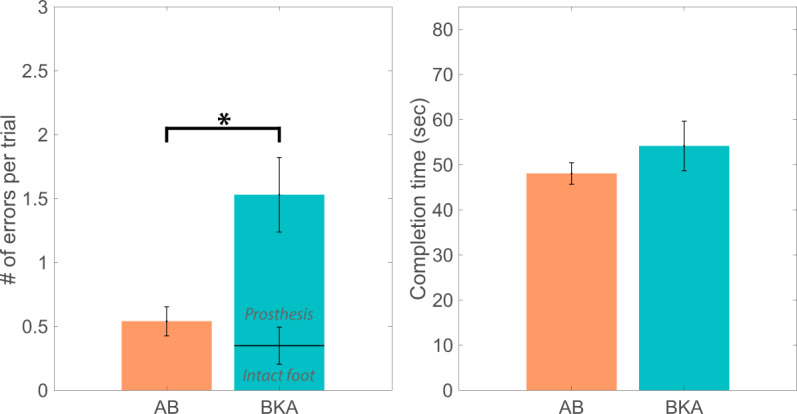
Task performance of able-bodied individuals and amputees. (**a**) The number of foot placement errors per trial is depicted for able-bodied individuals (‘AB’) and below-knee amputees (‘BKA’). For BKAs, the error rate bar plots are stacked to illustrate that the majority of the errors were made by the prosthetic foot rather than the intact foot. The black bracket indicates that the error rate between the participant groups was significantly different. **(b)** Trial completion times for able-bodied individuals and below-knee amputees were not significantly different.

Foot placement accuracy, but not trial completion time, was negatively affected by the presence of a lower-limb amputation. Able-bodied individuals made 0.5 ± 0.1 errors per trial (mean ± standard error) and BKAs made 1.5 ± 0.4 errors/trial (p = 0.006, Fig. 3a). For amputees, the majority of the errors were made with the prosthetic foot. To cross the ladder, able-bodied participants took 48.1 ± 2.4 s and BKAs took 54.2 ± 5.5 s (p > 0.05, Fig. 3b). We did not find any effects of gender, age, or height on these primary outcome measures.

**Figure 4:**
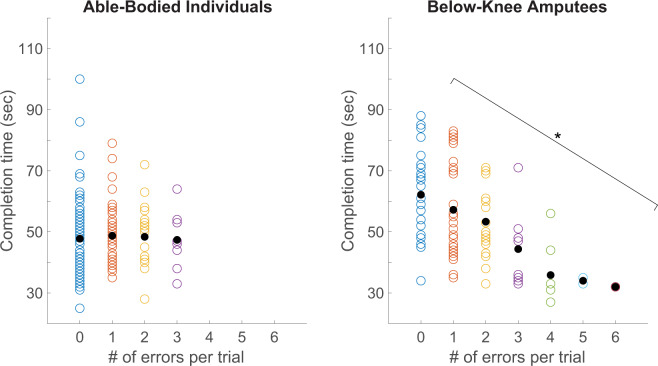
Speed-accuracy tradeoff for able-bodied individuals and amputees. Trial completion time is plotted against the number of errors per trial for able-bodied individuals (left) and below-knee amputees (right). A “speed accuracy tradeoff” is defined as an inverse relationship between completion time and error rate. The bracket indicates that there was a statistically significant relationship between the number of errors per trial and completion time for below-knee amputees (linear regression, p < 0.001), but not able-bodied individuals.

We confirmed that for BKAs, there was a speed-accuracy tradeoff with an inverse relationship between trial completion time and the number of foot placement errors. For BKAs, trials with a higher number of errors were typically completed in a shorter amount of time (p < 0.001, Fig. 4b). Interestingly, there was not a significant relationship between these variables for able-bodied individuals (Fig. 4a).

**Figure 5:**
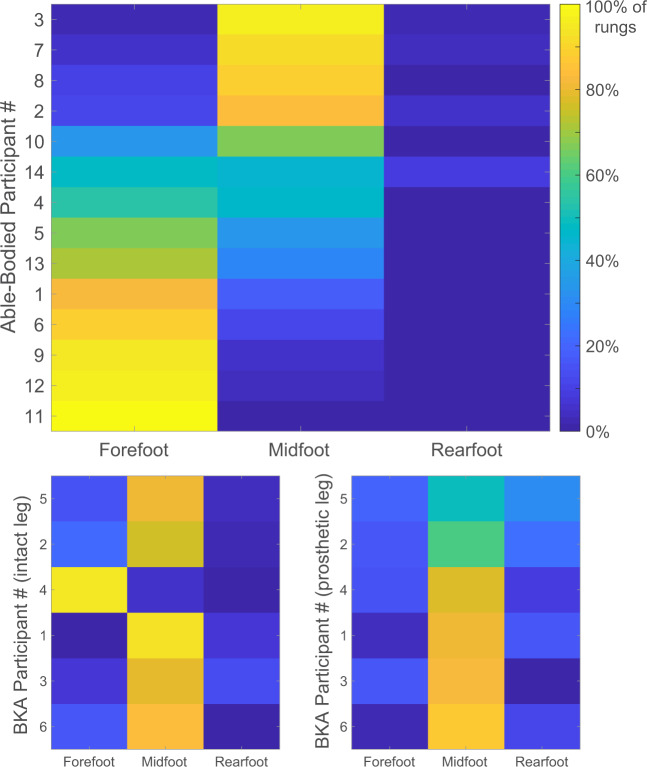
Region of the foot used to step on ladder rungs for able-bodied individuals and amputees. The 14 able-bodied participants most frequently used the forefoot to step on a ladder rung (top graph). The six BKAs primarily adopted a midfoot strategy for both limbs (intact leg on the bottom left, prosthetic leg on the bottom right). Yellow indicates that a foot region was used to step on 100% of the ladder rungs. For able-bodied participants and the prosthetic leg of BKAs, rows are sorted according to the midfoot activation percentage rather than participant number in order to more easily visualize trends.

Able-bodied individuals and BKAs differed in their foot placement strategy. Able-bodied controls most frequently stepped on ladder rungs with their forefoot (Fig. 5). Seven volunteers strongly preferred the forefoot (>60% of rungs), five volunteers preferred the midfoot (>60%), and two volunteers split steps almost evenly between the forefoot and midfoot. When stepping on a ladder rung with the prosthetic limb, BKAs most frequently landed on their midfoot. Out of all of the steps with the prosthetic limb from all BKA participants, 13% were on the forefoot, 72% on the midfoot, and 15% on the rearfoot. All BKAs, except for participant BKA04, also favored the midfoot with their intact leg. Out of all of the steps with the intact limb from all BKA participants, 25% of steps were on the forefoot, 70% on the midfoot, and 5% on the rearfoot. There was no relationship between the region of the foot used to step on each ladder rung and error rate or trial completion time (Supplemental Figure S1).

### Performance of BKAs while using the sensory neuroprosthesis

**Figure 6:**
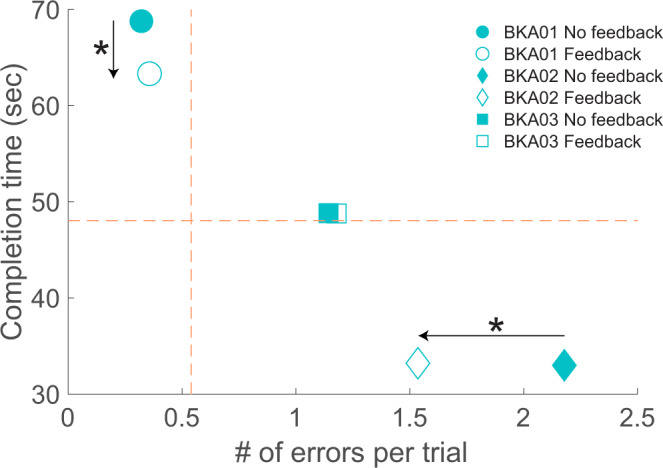
Speed-accuracy tradeoff for amputees using the sensory neuroprosthesis. In the “No feedback” condition, BKAs did not receive stimulation-evoked somatosensory feedback. In the “Feedback” condition, they received modulated feedback regarding plantar pressure from the sensory neuroprosthesis. The dashed orange lines denote the mean error rate and mean completion time for the able-bodied participants. The speed-accuracy tradeoff significantly improved for two out of three BKAs when the ladder test was performed while receiving feedback, as indicated by black arrows and stars.

**Figure 7:**
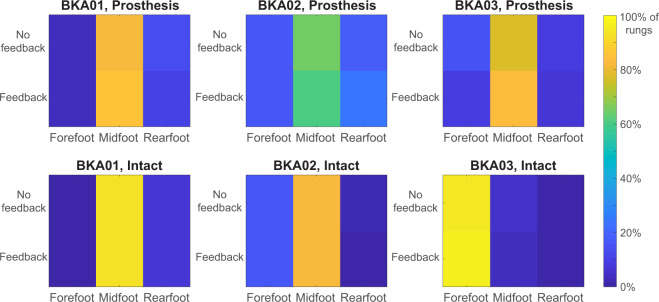
Region of the foot used by BKAs to step on ladder rungs when using the sensory neuroprosthesis. In the “No feedback” condition, BKAs did not receive stimulation-evoked somatosensory feedback. In the “Feedback” condition, they received modulated feedback regarding plantar pressure from the sensory neuroprosthesis. Foot placement strategy did not change for either limb during trials completed while using the sensory neuroprosthesis.

When amputees performed the task with the sensory neuroprosthesis, the tradeoff between speed and accuracy improved for two out of three BKAs (Fig. 6). The number of foot placement errors per trial significantly decreased for participant BKA02 (p < 0.001) and trial completion time decreased for participant BKA01 (p = 0.01). Participant BKA02 made an average of 2.2 ± 0.4 errors per trial without feedback, and only 1.6 ± 0.3 errors per trial when receiving feedback from the sensory neuroprosthesis. The decrease in overall error rate while using the sensory neuroprosthesis was predominantly caused by a decrease in errors made with the prosthetic foot (p = 0.02), not the intact foot. Participant BKA01 took 68.8 ± 2.0 s without stimulation-evoked sensory feedback and 63.3 ± 2.1 s with feedback. For participant BKA03, neither speed nor accuracy improved during trials with feedback. For all three BKA participants, foot placement strategy, i.e. the region of the foot used to step on each rung, did not change for either the prosthetic or intact limbs during trials performed using the sensory neuroprosthesis (Fig. 7).

## Discussion

Little is known about the impact of missing somatosensory information on lower-limb amputee locomotion. Most locomotor assessments do not directly assess the role of unilateral plantar sensation because they do not prevent compensation with other resources, such as vision. Therefore, we developed a novel ambulatory searching task based on prior animal studies. The blindfolded, horizontal ladder walking test approximates challenging real-world scenarios in a controlled setting. The ladder test mimics situations that pose a high fall risk, such as walking outside in the dark or carrying items that block the view of the ground. When able-bodied individuals and lower-limb amputees performed the test, differences in foot placement accuracy indicated that cutaneous plantar sensation is used to acquire action-relevant information during locomotion. We also demonstrated that a sensory neuroprosthesis that provides modulated feedback of plantar pressure can improve the performance of lower-limb amputees in challenging locomotor tasks.

### Comparison of able-bodied individuals and below-knee amputees

In the first half of the study, we confirmed that lower-limb amputees made more errors per trial than able-bodied individuals, but did not have different speeds. Though trial completion time is one of the most commonly used outcome measures in clinical assessments^41^, functional differences between amputees and able-bodied people may become more distinct when trial time is combined with error rate. We did not observe a significant relationship between foot placement strategy and accuracy, suggesting that differences in error rate between able-bodied individuals and BKAs could not be explained by foot placement strategy.

We also observed a speed-accuracy tradeoff for BKAs but not for able-bodied individuals. Although all participants focused their attention on completing the task, the able-bodied individuals had more compensatory tactile and proprioceptive inputs at their disposal to maintain accuracy even at faster speeds. These resources were lacking from the missing limbs of BKAs, who therefore had to slow down to maintain foot placement accuracy. Additional research could be conducted to fully map the relationship between completion time and error rate for able-bodied individuals by treating speed as a controlled independent variable, that is by enforcing specific speeds and measuring the associated accuracy. Overall, the established speed-accuracy tradeoff indicates that the horizontal ladder test could be used as a clinical tool for identifying locomotor deficits that get masked by sensory reweighting.

The number of errors made by the intact foot in amputees was qualitatively similar to the total number of errors made by both feet combined of able-bodied individuals, indicating that unilateral amputation affects the searching performance of both legs. This may be because amputees have trouble maintaining balance during single-leg stance on the prosthesis and have a lower balance confidence^1,42^, which could have caused them to rush through the act of searching with their intact foot. In future studies, it would be interesting for able-bodied participants to perform the task with cutaneous plantar sensation temporarily disrupted in one foot to further explore this phenomenon.

To step on a ladder rung, able-bodied individuals typically preferred to use the forefoot and amputees used the midfoot. The forefoot has a longer moment arm than the rearfoot with respect to the ankle joint, resulting in the highest ankle moment when load is applied to the forefoot^43^. Stepping with the forefoot, rather than the midfoot or rearfoot, allows the ankle to make corrective actions. While this was possible for able-bodied individuals, none of the BKAs in this study had volitional control over their prosthetic ankles. One of the BKAs explained that he chose a midfoot strategy because it was the “safest” location, i.e., the furthest from either end of the foot.

Interestingly, the vast majority of BKAs preferred to use their midfoot with the intact limb as well, which provides valuable insight into the principles of bilateral foot positioning. The BKAs adopted a strategy that maximized accuracy with the prosthetic leg and mimicked it with the intact leg, despite having a fully functional ankle. Bilateral coupling has been reported in other locomotor studies. When navigating stairs, unilateral lower-limb amputees use the same placement strategy for each foot^44^. Similarly, when encountering a sudden obstacle, unilateral lower-limb amputees have temporal delays in muscle activation in both the intact and residual legs^45^. The central integration of bilateral sensory inputs is critical for determining body position and organizing adaptive motor responses, particularly for maintaining balance and posture^46^. Moreover, lines of evidence suggest strong connections between the two legs in centralized motor control circuits^47^, which could predispose adoption of similar strategies between limbs. To gather more insight into this phenomenon, the horizontal ladder test could be repeated with individuals early in their post-amputation rehabilitation and with amputees who do not report gait automaticity (i.e., people that have to think about every step they take^48^).

### BKA task performance with somatosensory feedback

The speed-accuracy tradeoff improved for two out of three BKAs when the ladder test was performed while receiving feedback from their sensory neuroprostheses. BKA01 prioritized accuracy and BKA02 prioritized speed under all conditions, and somatosensory feedback from the neuroprosthesis improved whichever aspect was given the lower priority. For participant BKA01, error rate stayed the same and completion time decreased. For BKA02, completion time stayed the same and error rate improved. Participant BKA03’s performance on the horizontal ladder test remained unchanged when he used the sensory neuroprosthesis. As the most recent recipient of the implanted system, this could partly be due to his inexperience with sensory stimulation and the closed-loop neuroprosthesis.

BKA03 received his implant five months prior to performing the ladder test, and had participated in just 12 hours of sensory mapping experiments^24^ and one three-hour session using the closed-loop neuroprosthesis while walking on a treadmill. Therefore, to increase the participant’s experience with sensory stimulation, we provided BKA03 with a pre-programmed stand-alone external stimulator that could be utilized at home. Using the system at home, similar to mapping experiments in the laboratory, he was able to deliver stimulation to selected electrode contacts, which elicited sensations in his missing foot. Although the stimulation level did not correspond to foot-floor interactions, we hypothesized it would help the participant become more familiar with elicited sensations and gain more confidence in determining the location and modality of the percepts. After three weeks, we asked him to repeat the ladder test. Although he did not report any changes in the location, modality, or intensity of stimulation-evoked somatosensation, his trial completion time for the ladder test was significantly reduced when he used the sensory neuroprosthesis (Supplemental Figure S3, p < 0.001). It is possible that as he uses the sensory neuroprosthesis more often, he will learn to incorporate that feedback during challenging locomotor tasks. To maximize rehabilitative and functional benefits for all BKAs, it might be best for amputees to wear the sensory neuroprostheses outside of the laboratory for days at a time. This added time in different environmental contexts could facilitate gait re-training and the integration of stimulation-evoked somatosensation with other sensory resources.

Finally, the region of the foot used by BKAs to step on a ladder rung did not change during trials performed with the sensory neuroprosthesis. As previously mentioned in the Methods, we suspected that foot placement strategy would be influenced by biomechanics, volitional ankle control, and cutaneous plantar sensation. The lack of a change in foot placement strategy while using a sensory neuroprosthesis indicates that in this context, foot placement strategy was not affected by cutaneous sensation. Additionally, as previously mentioned, there was not a significant relationship between foot placement strategy and accuracy. In other words, biomechanics and volitional ankle control do not strongly influence foot placement accuracy in this task. The differences in foot placement accuracy between able-bodied individuals and amputees are likely caused by another factor, such as the presence of cutaneous plantar sensation.

### Limitations

As this was a novel task, the present study had some limitations. Our findings could be more generalizable if they are repeated in a larger group of people with more diverse demographics in age and sex, as well as amputation etiologies. A direct comparison of amputees to age- and gender-matched controls would strengthen our argument that higher error rates are a result of amputation, rather than older age. To date, only four BKAs have received our novel sensory neuroprosthesis, and one of these individuals was unable to perform the task due to unrelated health concerns. Additionally, all participants wore their own closed-toe shoes in order to better capture their typical ambulation strategy. Although prescribing a standard set of footwear would have eliminated a potential source of inter-participant variability, particularly with respect to which region of the foot was placed on a ladder rung, it may have compromised familiarity and comfort which might have also affected the results. Finally, the handrail was only available on one side of the participant during each trial. Location of the unilateral rail was fixed, which means it alternated between the dominant/non-dominant hand and missing/intact leg as participants completed trials in different directions. This may have increased task difficulty and also could have influenced strategy, although we did not observe any interactions with direction.

### Future work

In future repetitions of the horizontal ladder test, it would be interesting to determine if foot placement accuracy or strategy is dependent on footwear or the presence of dual handrails. Clinical measures of balance confidence^49^ could also be assessed prior to application of the assessment and utilized as a covariant in future statistical analyses to determine how the fear of falling impacts ambulatory searching. Additionally, it would be interesting to conduct the ladder test with fallers and non-fallers to determine if it could be used as a clinical balance tool for those who find current clinical tests too easy, but still have a significant history of falls.

## Conclusion

This study probed the role of cutaneous plantar sensation in locomotion using a novel ambulatory searching task. We examined how people with unilateral lower-limb amputations adapt to the loss of plantar sensation, and how that sensation is integrated into the control of bipedal locomotion. The higher error rate in BKAs compared to able-bodied individuals indicated that cutaneous plantar sensation plays a major role in optimizing ambulatory searching. This notion was corroborated in the BKAs who performed the task with sensory neuroprostheses and demonstrated an improvement in speed-accuracy tradeoff. Our results suggest that the body is able to integrate and utilize PNS-evoked somatosensory inputs appropriately in challenging locomotor tasks. These findings indicate that the information related to interactions between the prosthetic foot and the ground provided by sensory neuroprostheses can improve complex locomotor function in lower-limb amputees.

## Supplementary information

Supplementary information.

## Data Availability

The datasets generated during the current study are available from the corresponding author on reasonable request.
