# Author Correction: Ambulatory searching task reveals importance of somatosensation for lower-limb amputees

**DOI:** 10.1038/s41598-022-17833-5

**Published:** 2022-08-16

**Authors:** Breanne P. Christie, Hamid Charkhkar, Courtney E. Shell, Christopher J. Burant, Dustin J. Tyler, Ronald J. Triolo

**Affiliations:** 1 Department of Biomedical Engineering, Case Western Reserve University, Cleveland, OH USA; 2 Louis Stokes Cleveland, Department of Veterans Affairs Medical Center, Cleveland, OH USA; 3 Department of Biomedical Engineering, Lerner Research Institute, Cleveland Clinic, Cleveland, OH USA; 4 School of Nursing, Case Western Reserve University, Cleveland, OH USA

Correction to: *Scientific Reports* https://doi.org/10.1038/s41598-020-67032-3, published online 23 June 2020

The original version of this Article contained an error in Figure 1, where the width dimension of the testing apparatus was incorrect.

The original Figure 1 and accompanying legend appear below.

**Figure 1:**
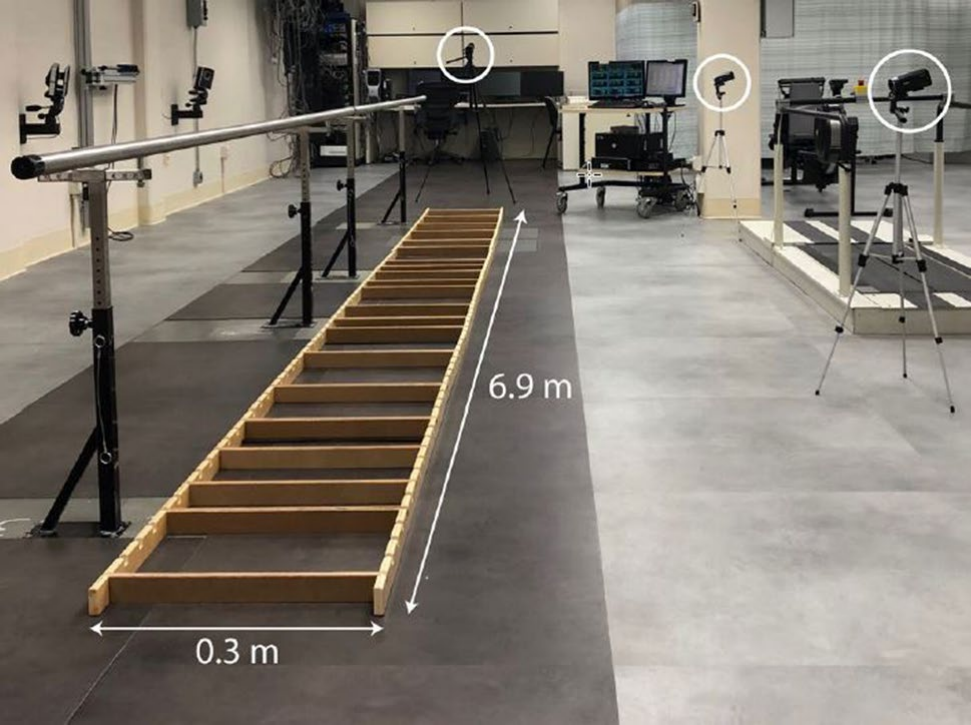
Experimental setup. Able-bodied volunteers and below-knee amputees performed a horizontal ladder rung walking test while blindfolded. Ladder rungs were randomly spaced 19, 28.5, 38, or 47.5 cm apart and the arrangement changed after every trial. Participants used a single handrail that ran alongside the ladder for support. Videos were recorded with three cameras, two alongside the ladder and one at the end.

As a result, in the Materials and methods, under the sub-heading ‘Experimental design’,

"The testing apparatus was constructed out of wood and measured 6.9 m long by 0.3 m wide, as depicted in Fig. 1."

now reads:

"The testing apparatus was constructed out of wood and measured 6.9 m long by 0.59 m wide, as depicted in Fig. 1."

The original Article has been corrected.

