# Supplementary material for: Ambulatory searching task reveals importance of somatosensation for lower-limb amputees
Source: Sci Rep. 2020 Jun 23;10:10216. doi: 10.1038/s41598-020-67032-3 (PMC7311393; doi:10.1038/s41598-020-67032-3)

Ambulatory searching task reveals importance of somatosensation for lower-limb amputees

Breanne P. Christie*

Hamid Charkhkar

Courtney E. Shell

Christopher J. Burant

Dustin J. Tyler

Ronald J. Triolo

Supplemental Table S1: Demographics of the able-bodied individuals who participated in the horizontal ladder test.

Participant ID	Age (years)	Height (m)	Gender
AB-01	31	1.73	Female
AB-02	19	1.68	Male
AB-03	20	1.88	Male
AB-04	18	1.75	Female
AB-05	25	1.78	Female
AB-06	44	1.65	Male
AB-07	19	1.65	Female
AB-08	31	1.73	Female
AB-09	26	1.75	Female
AB-10	58	1.52	Female
AB-11	27	1.68	Male
AB-12	39	1.70	Male
AB-13	52	1.65	Female
AB-14	66	1.73	Male

Supplemental Table S2: Demographics of the below-knee amputees who participated in the horizontal ladder test. ESAR: energy-storage-and-return.

Participant ID	Age (years)	Height (m)	Gender	Amputated limb	Cause of amputation	Years since amputation	Prosthesis type
BKA01	70	1.73	Male	Left	Trauma	49	ESAR
BKA02	56	1.65	Male	Right	Trauma	11	Active
BKA03	57	1.83	Male	Right	Trauma	9	ESAR
BKA04	59	1.68	Male	Right	Vascular	5	ESAR
BKA05	58	1.78	Male	Left	Cancer	4	ESAR
BKA06	40	1.85	Male	Right	Trauma	2	ESAR

Supplemental Figure S1: Relationship between foot placement accuracy and strategy for able-bodied individuals and amputees. Foot placement strategy is represented by the percentage of trials in which the participant stepped on a ladder rung using their forefoot. The results of a linear regression showed no correlation between error rate and the region of the foot used to step on the ladder rung.

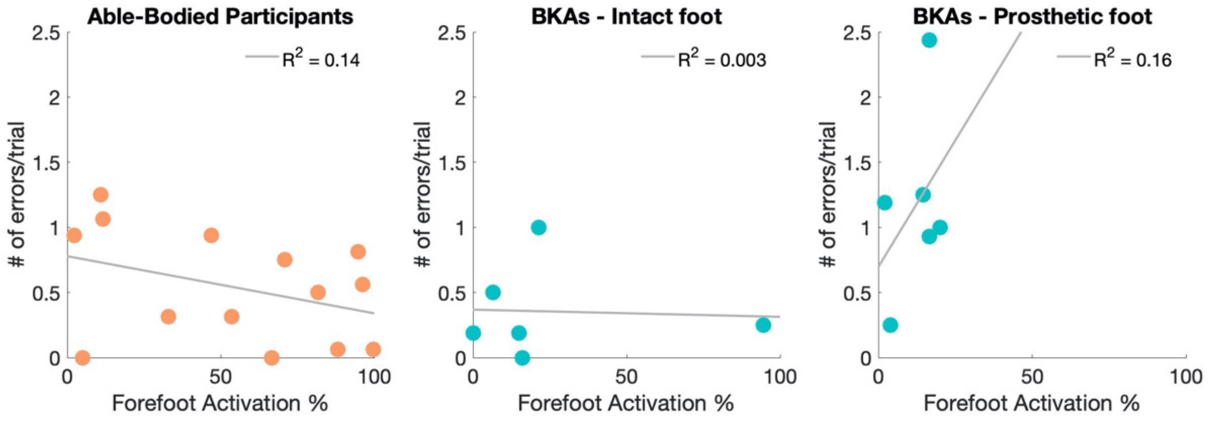

Supplemental Figure S2: Relationship between trial completion time and foot placement strategy for able-bodied individuals and amputees. Foot placement strategy is represented by the percentage of trials in which the participant stepped on a ladder rung using their forefoot. The forefoot percentages of the intact and prosthetic foot were averaged for this analysis. The results of a linear regression showed no correlation between trial completion time and the region of the foot used to step on each ladder rung.

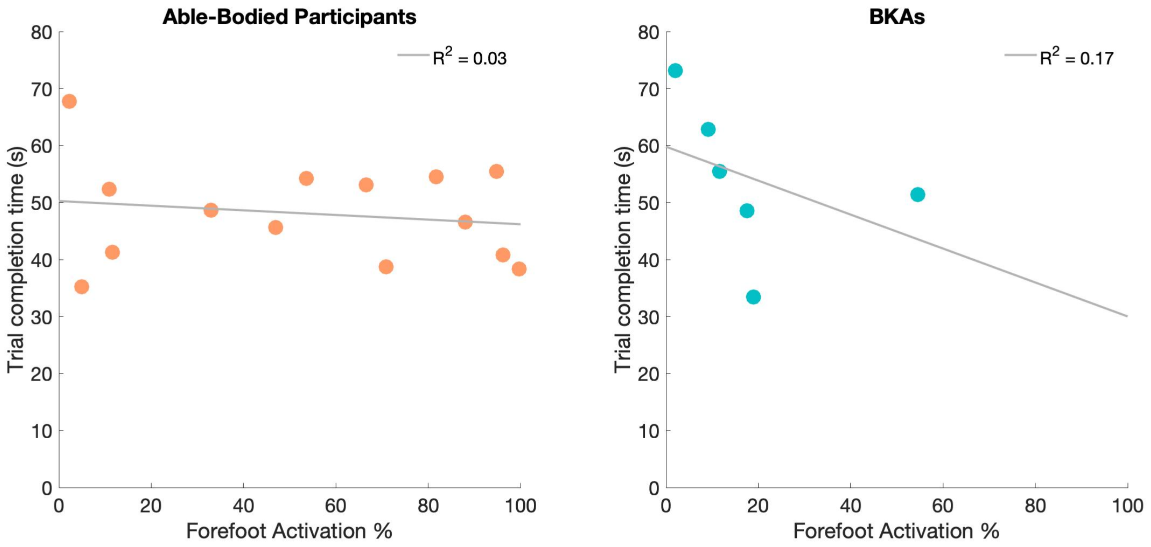

Supplemental Figure S3: Task performance across testing sessions. 'Session #' refers to the number of experimental sessions in which the participant performed the ladder test. Brackets indicate statistically significant differences between the "No feedback" and "Feedback" conditions. Each session included at least 10 trials/condition, and sessions occurred approximately 3-7 weeks apart. In sessions #1-2, participant BKA03 did not perform the task

significantly better while receiving sensory feedback. After session #2, this participant received a pre-programmed stimulator that could be utilized at home to gain familiarity with evoked somatosensory percepts. In session #3, BKA03 performed the task significantly faster during trials with sensory feedback (one-tailed paired t-test, $p<0.001$). Moreover, participant BKA01 had used a sensory neuroprosthesis for over three years and was able to effectively utilize sensory feedback starting in the first experimental session. Though not statistically significant, both completion time ($p=0.07$) and error rate ($p=0.08$) trended towards lower values during trials with sensory feedback in session #1. There was also a learning effect for the ladder task itself. As sessions progressed and participants acquired more practice, completion time decreased for BKA01 (linear regression across "No feedback" sessions, $R^2=0.88$) and error rate decreased for BKA03 ($R^2=0.96$).

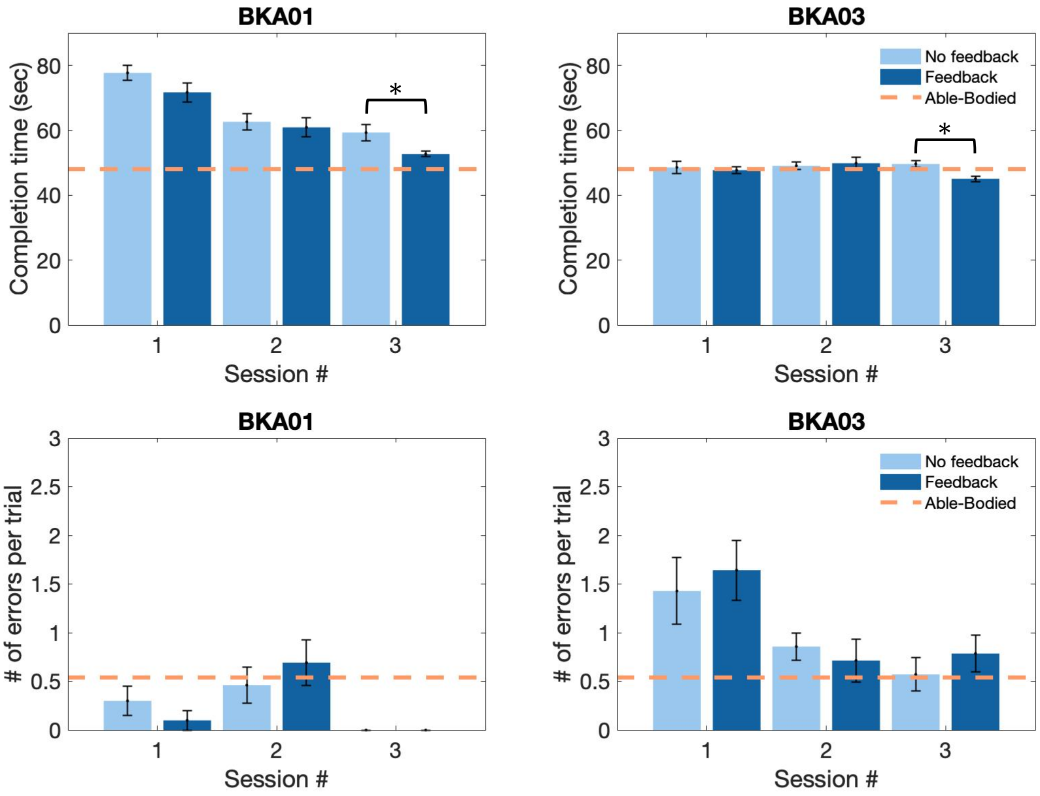

Supplement: Supplementary file 1 — Supplementary information. [file 41598_2020_67032_MOESM1_ESM.pdf]
